# Relationships between Self-Talk, Inner Speech, Mind Wandering, Mindfulness, Self-Concept Clarity, and Self-Regulation in University Students

**DOI:** 10.3390/bs14010055

**Published:** 2024-01-15

**Authors:** Famira Racy, Alain Morin

**Affiliations:** 1 Independent Researcher, MA Psychology, Calgary, AB T3E 6K6, Canada; 2 Department of Psychology, Mount Royal University, 4825 Mount Royal Gate SW, Calgary, AB T3E 6K6, Canada; amorin@mtroyal.ca

**Keywords:** inner speech, self-talk, mind wandering, mindfulness, self-concept clarity, self-regulation, self-processes

## Abstract

In this study, the researchers explored novel relationships between the self-related processes of self-talk, inner speech, self-concept clarity, mindfulness, mind wandering, and self-regulation. Using self-report questionnaires (*n* = 227), we found a small positive association between inner speech use and mind wandering, as well as a medium positive association of mindfulness with self-regulation, in accordance with claims made in the literature. We found a medium positive relationship between mindfulness awareness and inner speech responses, potentially because mindfulness awareness represents an active state of self-focus, requiring verbal self-directed probes. Conversely, the correlations between reports of inner speech use and mindfulness acceptance were medium and negatively associated, perhaps because self-acceptance is a less active process that does not require as much self-directed speech as acquiring self-awareness, or perhaps self-acceptance consists of silencing the mind. Furthermore, the mindfulness-acceptance responses were negatively associated with mind wandering but positively correlated with self-concept clarity and self-regulation (all significant and of medium strength). Another noteworthy result was that mind wandering was negatively correlated with self-concept clarity and self-regulation, in accordance with the view that mind wandering represents a self-escape mechanism and thus impedes the transparency of one’s self-view and efforts at controlling oneself. This study pieces together what has been presented in the literature, examining variables that are typically studied in isolation. Further, these results have implications for the future study of self-regulation in that self-focused attention, self-acceptance, and self-concept clarity may be mediators on the paths between self-talk or inner speech use and self-regulation.

## 1. Introduction

It is becoming increasingly evident that the study of the self should include—if not emphasize—an examination of how its various aspects relate to one another before assuming that specific aspects alone interact or affect one another [1]. The self is usually understood as being multidimensional in nature, made up of both conscious and unconscious levels, and informed by the observations of others [2]; it includes all conceivable private and public aspects that make up who a person is, such as thoughts, emotions, goals, values, sensations, memories, traits, attitudes, physical attributes, behaviors, and skills. The self represents a highly dynamic system constituted of social, cognitive, emotional, motivational, and neurological dimensions (e.g., [3,4,5,6]). Indeed, the self does not represent a unitary concept [7]. Morin ([1], Figure 1) illustrated several existing and potential connections between self-related constructs, showing this complexity of the self. Since then, some self-related processes such as inner speech have gained more in-depth conceptualization, which we take as justification for exploring relationships further with empirical investigation. In particular, we are interested in bridging the common study of self-talk and self-regulation with the specific study of inner speech, along with mind wandering, mindfulness, and self-concept clarity. Each concept has its own body of literature representing it, but to our knowledge, these variables have not been assembled in one single study.

### 1.1. Self-Talk and Self-Regulation from the Vygotskian Perspective

The social nature of cognitive development is intimately woven into the self-regulatory function of self-talk. Self-talk is defined as self-directed speech emitted out loud [8]. Researchers studying self-talk tend to agree with the Vygotskian view (e.g., [9]) that developing children witness social speech (e.g., parents talking to each other), then mimic and practice social speech by uttering to themselves out loud (private speech, e.g., imaginary friends) and engaging in social speech with others. The development of inner speech (silent self-directed speech) around age seven is thought to happen via mental internalization and the condensation of social and private speech. From this perspective, one can easily take the stance that inner speech is social in nature. For example, one may experience inner speech in phenomenologically varied ways, such as replaying or imagining social conversations or having conversations and dialogue with oneself (e.g., [10]). 

Self-talk measurement can have strong self-regulatory aspects, which is also in line with the Vygotskian perspective (e.g., [8,11]). Altering one’s behavior, resisting temptation, changing one’s mood, making a choice, and filtering irrelevant information are clear examples of self-regulation [12]. There is a large body of literature which supports the importance of self-regulatory speech-for-self in children (e.g., [9,13,14]) and adults (e.g., [15]). It is completely natural and healthy to experience self-talk, even in a conversational fashion, in order to control oneself, as Alderson Day and Fernyhough [16] showed in their thorough review including self-talk phenomenology and self-regulation. A recent self-talk model [17] discusses how “goal-directed self-talk” as well as “educational and strategic self-talk interventions” can be related to variables such as task difficulty, emotions, behavior, performance, metacognition, and concentration, all of which can be seen as self-regulatory in nature.

Even throughout adulthood, people experience self-talk internally or use it out loud to instruct themselves, encourage or criticize themselves, rehearse, and much more. It may be that some forms of self-talk are more helpful for self-regulation than other forms. In terms of questionnaire-based studies, self-talk for self-regulation responses had weak-to-moderately strong relationships with various inner speech measures in one study [18] and with types of inner dialogues (e.g., identity, rumination) in another study [19]. In essence, there is a connection between overt and covert self-talk, and they both share self-regulatory functions, although they cannot be equated because the development, phenomenology, and functional range of covert and overt self-talk vary [20]. Even though there are other forms of experimental and neurobiological evidence for the usefulness of self-talk in self-regulation, conceptualizations as well as various questionnaire tools are still in the development stages. Furthermore, researchers are exploring a variety of other self-processes that may be involved in the pathways to self-regulation. Therefore, understanding the relationships between self-talk or inner speech and self-regulation requires a variety of tools and an exploration of what other self-processes might be involved.

### 1.2. Self-Concept Clarity, Mindfulness, and Mind Wandering

In addition to the relationship between self-talk and self-regulation, researchers have also found links between self-directed speech and self-concept clarity, mindfulness, and mind wandering (e.g., [21,22]). Self-concept clarity entails the clarity and stability of one’s self-concept [23] and having a clear sense of self allows one to form more precise and realistic plans to achieve one’s goals (e.g., [24]), and a lack of self-concept clarity may inhibit self-control [25]. Self-talk may be involved in how people conceptualize the self (e.g., [26,27]). Conversely, a group of researchers found modest negative relationships between some phenomenological varieties of inner speech (but not others) and self-concept clarity in a clinical sample, with some differences and some similarities between the clinical and non-clinical groups on inner speech [21]. When examining this relationship with different measures, university samples (instead of clinical), and a very specific, dialogical variety of inner speech, Oleś and colleagues [19] reported that higher self-concept integration was associated with less frequent internal dialogical activity. It may be that age or development, phenomenology, and, at the minimum, cultural variations, play into both inner speech manifestations and self-concept clarity, as reported above; but another possibility is that people with a clear sense of self do not need to talk to themselves extensively for self-identity purposes. 

Mindfulness is usually defined as a non-judgmental focus on the self in the present moment [28]. Grzybowski [29] found that positive self-talk increased together with trait mindfulness, and later found that self-regulatory self-talk increased together with some (but not all) aspects of trait mindfulness [30]. The study also showed small but significant negative relationships between a non-judging facet of mindfulness and self-talk for self-management, self-criticism, and social assessment. Perhaps the need to manage and criticize the self and carry out social assessments via self-talk decreases as non-judgement in the present moment increases. There is also evidence from neurological studies, such as one by Riegner and colleagues [31], who found that mindfulness induced weak connectivity between brain areas known to sustain self-referential processing (e.g., the prefrontal cortex), presumably including self-directed speech. 

It is also common to distinguish between mindful awareness (being aware of one’s internal and external surroundings) and mindful acceptance (non-judgmentally accepting present-moment experiences [32]). Mindful awareness might represent a more active state of self-focus requiring verbal, self-directed probes than mindful acceptance. Conversely, mindful acceptance in the present moment may entail quieting judgmental, self-critical inner speech. Considering this logic and the aforementioned mixed relationships between mindfulness and self-talk, it may be that self-talk is involved in mindfulness in different ways, depending on which aspects of self-talk or mindfulness are being measured. 

Reports of mindfulness as an enabler of self-regulation, especially in an emotional regulation capacity, have been peaking [33,34,35]. However, this is not always the case (although sometimes it is) for mind wandering, which represents random, off-task thoughts experienced when one is engaged in attention-demanding tasks [36]. While mind wandering can interfere with task focus, perhaps especially when there are less cognitive tasks and more complexity [37], it is important to note that mind wandering is not always a bad thing—it might also have something to do with self-communication [38,39], may take the form of verbal conversations with oneself [40], and may even contribute to creativity [41]. Even as such, some seek to eliminate mind wandering [42] and increase self-control [43] using mindfulness.

In essence, putting these pieces together makes it clearer that self-talk probably has a role to play in all of these processes. For example, people may talk to themselves when they are seeking or forming self-concept clarity, when they are gaining awareness of their experience, or when they are distracted from a task. People who use less self-talk or inner speech, are unsure about who they are, or tend to mind wander might also have difficulty with self-regulation. Mindfulness and self-concept clarity are probably helpful for self-regulation and self-talk, whether internal (inner speech) or external (private speech) speech is used.

### 1.3. The Present Study

Self-talk and inner speech are theoretically paramount to self-regulation from a Vygotskian viewpoint, but researchers are also keen to understand how other self-processes are involved in self-regulation. Further, there are mixed results regarding the connections between self-talk and self-regulation, and questionnaire use as well as conceptualizations in the field are still in development. This means further research is required to advance understanding in this area. Finally, researchers have examined some relationships between these variables, but, to our knowledge, not in a systematic fashion as is done here. Therefore, the purpose of this study is to undertake this basic starting point by investigating relationships between self-talk, inner speech, self-concept clarity, mind wandering, mindfulness, and self-regulation; we hope to replicate some results already found in the literature. In sum, we are investigating a series of hypotheses regarding these relationships, as illustrated in Table 1. 

## 2. Methods

### 2.1. Participants, Procedure, Data Screening, and Analysis

The participants were 227 consenting Mount Royal University students in Canada (*M* = 21.1 years of age; *SD* = 5.10; 180 females made up 79.3% of the sample). Students were recruited using SONA, a common tool for managing university research studies, and they completed the questionnaire package in a classroom setting. Students also completed a Human Research Ethics Board (HREB—101097) approved package for credit in the Psychology Program. Upon visual inspection of the data, we entirely removed all reports containing missing chunks of data or reports that appeared to be filled in inaccurately (e.g., reporting all similar responses on all items even when some items were reversed). We used SPSS v. 24 to conduct normality and outlier analyses, as well as Pearson’s correlations and to obtain reliability coefficients. We used the following guidelines to interpret correlation coefficient magnitudes in the context of psychology research: correlation coefficients ranging from 0.10 to 0.29 are “small”, those from 0.30 to 0.49 are “medium”, and those greater than 0.50 are “large” [47]. 

### 2.2. Measures

#### 2.2.1. Self-Talk Scale (STS) 

The 16-item STS [8] uses a 6-point Likert scale (from *1 = never* to *6 = always*) to measure the self-reported frequency of self-talk in non-clinical populations. The STS is commonly used (e.g., [48], more recently in performance contexts (e.g., [49,50]) because of its factors around self-regulation: (1) social assessment (e.g., “I talk to myself when I review things I’ve already said to others”), (2) self-criticism (e.g., “… when I feel discouraged about myself”), (3) self-management (e.g., “… when I’m mentally exploring a possible course of action”), and (4) self-reinforcement (e.g., “… when I need to boost my confidence that I can do something difficult”). Racy et al. [22] reported acceptable reliability for this measure in their sample (α = 0.88). Brinthaupt et al. [8] reported test–retest reliability (r = 0.66) and preliminary construct validity, for example, using a measure of automatic positive self-statements (r = 0.36 with STS reinforcement) and negative self-statements (r = 0.35 with STS self-criticism). 

#### 2.2.2. General Inner Speech Questionnaire (GISQ)

The 57-item, 6-point (from *0 = never* to *5 = all the time*) GISQ [22] was built for general use in non-clinical populations. The GISQ asks what people think they talk to themselves ‘about’, what they use inner speech ‘in order to’ do, and ‘when’ they use inner speech, with an overall score representing the perceived frequency of inner speech content and functions in general. Items include contexts of “self-reflection” (e.g., “I talk to myself about who I am”), “cognition” (e.g., “I talk to myself in order to problem-solve”), and “activities” (e.g., “I talk to myself when driving”). Using a total score, the GISQ is reliable (α = 0.95) and relates to the self-regulatory STS (r = 0.64) by Brinthaupt and colleagues [8], showing stronger reliability and concurrent validity than other inner speech measures [18]. 

#### 2.2.3. Mind Wandering Questionnaire (MWQ) 

The 5-item MWQ [42] uses a 6-point Likert scale (*1 = almost never* to *6 = almost always*) to assess the trait-like frequency of task-unrelated thoughts (e.g., “I find myself listening with one ear, thinking about something else at the same time”). The authors established validity using the relationships between mind wandering measured via thought-sampling and performance on a working memory task. The MWQ has acceptable reliability (e.g., α = 0.85 [43]; α = 0.84 [22]) and has been used in various clinical samples such as in relation to attention deficit disorder and obsessive compulsive disorder [51]. The MWQ has also been used in various cultures and nonclinical samples, for example, in relation to mindfulness, metacognition, self-control [42], social influence, anxiety, and task performance [52].

#### 2.2.4. Philadelphia Mindfulness Scale (PHLMS) 

The 20-item, two-factor PHLMS [32] uses a 5-point scale (from *0 = never* to *4 = very often*) to distinguish between the self-reported frequency of present-centered awareness (e.g., “I am aware of what thoughts are passing through my mind”) and acceptance (e.g., “There are aspects of myself I don’t want to think about”, reverse-scored). The PHLMS performs with moderately strong predictive ability (e.g., [43,53]) and reliability in non-clinical samples (e.g., α_Awareness_ = 0.75; α_Acceptance_ = 0.75 [33]). The PHLMS is used often in the study of self-related processes such as mind wandering, metacognition, and performance. Using the subscales yields strikingly different results than using the total score alone (e.g., [54]).

#### 2.2.5. Self-Concept Clarity Scale (SCCS) 

The 12-item unidimensional SCCS [23] uses a Likert scale (*1 = strongly disagree*; *5 = strongly agree*) to measure agreement with statements about the clarity and stability of one’s self-concept, using mostly reverse-scored items such as “My beliefs about myself often conflict with one another” or “Sometimes I feel that I am not really the person that I appear to be.” The test–retest coefficients were between 0.70 and 0.79 with a strong average reliability coefficient (α = 0.86). Temporal stability and validity were established, but Campbell and associates [23] warned that cultural variability also plays a role in interpreting self-concept clarity results. 

#### 2.2.6. Self-Regulation Questionnaire (SRQ) 

The 63-item SRQ [55] uses a 5-point scale (from *1 = strongly disagree* to *5 = strongly agree*) to assess a non-clinical, self-reported, self-regulation capacity (e.g., “I think a lot about how I’m doing”). Validity was established using measures of alcohol dependency, punctuality, and impulsiveness. The measure is designed to tap regulatory processes such as information input, evaluation, perceiving discrepancies, triggering change, searching for ways to reduce discrepancies, planning change, implementing change, and goal progress evaluation—scores on which the authors recommend summing to yield a total score to represent overall self-regulatory capacity. The internal consistency of all the items was high (α = 0.91 [55]). Other researchers have proposed or extracted different models for specific contexts like academic achievement (e.g., [56]).

## 3. Results 

Of the 227 participants, 180 were female and 47 were male (*M* = 21 years old). Due to the overwhelming number of females in the sample, gender comparisons were not made. The participants rarely reported an absence of inner speech or self-talk; they reported more self-talk for self-criticism (*M* = 18.33) and social assessment (*M* = 18.30) than reinforcing self-talk (*M* = 15.69), with the most frequent endorsements occurring for self-management (*M* = 19.9 out of a possible total of 20 on the STS). They also reported more mindful awareness (*M* = 33.05) than mindful acceptance on the PHLMS (*M* = 23.21) and proportionally endorsed self-concept clarity (SCCS) less often than mind wandering (MWQ), on average. Although the participants reported frequent self-managing self-talk, they did not endorse self-regulation (SRQ) as often, on average. Table 2 shows the descriptive statistics, distribution characteristics, and reliability coefficients for all the measures. The reliability analyses revealed a spread of coefficients, with the GISQ at the top end and the SRQ at the lower end (*r* = 0.95–0.71). 

The correlational analyses (Table 3) revealed concurrent validity between the GISQ and the STS (*r* = 0.64). The self-regulation, self-criticism, self-management, and social assessment subscales of the STS were positively correlated with the GISQ scores, ranging from medium to large associations (from *r* = 0.41 to 0.55). The analyses also showed significant positive relationships between self-talk and mindful awareness, ranging from small to medium associations (*r* = 0.14, *p* < 0.05 to 0.31, *p* < 0.01) and insignificant to significant negative relationships between self-talk and mindful acceptance (*r* = 0.03 to −0.38, *p* < 0.01). Further, the results showed insignificant to small significant positive relationships between self-talk and mind wandering (*r* = 0.02 to 0.26, *p* < 0.01), insignificant to medium significant negative relationships between self-talk and self-concept clarity (*r* = 0.07 to −0.37, *p* < 0.01), and mixed small relationships between self-talk and self-regulation (*r* = −0.16, *p* < 0.05 to 0.21, *p* < 0.01). Finally, the results indicated significant medium positive relationships between self-concept clarity and self-regulation (*r* = 0.48), as well as between mindfulness and self-regulation (*r* = 0.43, *p* < 0.01), and a significant medium negative relationship between mind-wandering and self-regulation (*r* = −0.48; *p* < 0.01). 

## 4. Discussion

The eight main hypotheses of this study were largely supported by our results (Table 4). The more recent GISQ measure of inner speech [22] appeared to have construct validity. The people who reported using self-talk and inner speech also endorsed mindful awareness [30], as well as mind wandering [46], as expected if verbal thought plays a role in these processes. On the reverse side, the endorsements of self-talk and mindful acceptance, as well as self-talk and self-concept clarity varied together in opposite directions, as expected if mindful acceptance shuts down unwanted verbal thoughts [30] or if one does not need to verbally self-inquire about one’s self-concept [21]. Self-talk also shared mixed relationships with self-regulation, which is to be expected from the Vygotskian view [9] that inner speech serves self-regulatory functions; however, our results show that self-critical inner speech probably does not strengthen self-regulation, which makes sense if criticism is not constructive toward how one thinks, acts, or behaves.

Digging into the details, the participants who reported more inner speech on the GISQ and more self-talk on sub-scales of the STS (except for reinforcing self-talk) also reported more mind wandering on the MWQ. That is, self-talk in general (but perhaps not self-talk for self-reinforcement) as well as inner speech may be involved in mind wandering (e.g., [46]). It is reasonable to assume that a portion of freely generated thoughts not related to an ongoing task take the form of internal verbal conversations with oneself, perhaps in a frustrating capacity or perhaps for creativity or insight [40,46].

All the self-talk and inner speech responses were positively related to the PHLMS mindful awareness subscale responses, which makes sense if inner speech and self-talk help to gain information about the self (self-awareness [27]), as well as help to make verbal observations about one’s immediate environment when being mindful. On the other hand, the responses on the GISQ for inner speech and on the subscales of the STS (except for reinforcing self-talk) were negatively related to those on the PHLMS mindful acceptance subscale. The same pattern was found with the relationships between the self-talk/inner speech scales and the SCCS for self-concept clarity. These findings potentially mean that people who are more self-accepting and who have more self-concept clarity also experience less self-critical, social-assessing, and managing self-talk and inner speech, and less need for reinforcing self-talk (see [21]). If mindful acceptance is an opposite yet complementary process to mindful awareness (e.g., accepting the information that was obtained in awareness), then it makes sense to see acceptance associated with less inner speech and self-talk, as observed here, consistent with the conception that accepting self-focus in the present moment quiets the inner voice [30]. The same principle might apply to self-concept clarity—the clearer the self-concept, the less of a need to talk to oneself for clarity [21].

The responses on three out of the four STS subscales (but not the self-management subscale) significantly related in mixed directions to the responses on the SRQ for self-regulation, while the responses on the GISQ for inner speech did not significantly relate to those on the SRQ. These results possibly mean that these measures do not capture self-regulation in the context of making change or that people use more reinforcing self-talk and less of other self-talk or inner speech when they are self-regulating. The STS is meant to capture self-regulatory self-talk more so than the GISQ for inner speech [8,22]. The fact that there is no relationship between managing self-talk and self-regulation is puzzling given that managing self-talk includes items such as “I need to figure out what I should do or say”. Besides measurement issues, perhaps there is a third (or more) variable influencing the relationship between inner speech and self-regulation (see [57]). Taken together, these findings show that one should not jump to conclusions when interpreting these results; for example, one should not assume that practicing mindfulness will automatically reduce one’s inner speech or that having more self-talk automatically means one has more self-regulatory capacity; rather, the nature of these constructs and their relationships are more complex.

The responses of increased mindful awareness on the PHLMS were also significantly related to the reports of increased self-regulation, as expected, since people tend to practice mindfulness to obtain more control of their experiences [34,35]. Further, the reports of mindful acceptance were negatively related to inner speech and mind wandering while being positively related to self-concept clarity and regulatory capacity for making change. These findings suggest that mindfulness, when used with both awareness and acceptance, is potentially involved in both raising and lowering inner speech and self-talk without raising mind wandering and may also have something to do with capacity to change. 

Besides being positively associated with inner speech and self-talk (but not regulatory self-talk) and negatively associated with mindful acceptance, mind wandering was also negatively associated with self-concept clarity and self-regulation. While these results could point to mind wandering as perhaps being related to an unclear view of self [58], it is impossible to know the directions of these relationships or to know whether mind wandering is a hindrance, an escape, or an enjoyable experience [59,60]. Self-concept clarity was also positively associated with self-regulation, suggesting that having a clear sense of self might have something to do with the capacity to make change in one’s life or vice versa, which supports Markus’ [24] seminal claim that knowing who oneself is helps one behave in ways to achieve one’s goals. 

## 5. Conclusions, Limitations, and Future Research

This study brings together self-processes that are typically studied independently, replicating and extending some of what has been presented in the literature. Our findings show that inner speech and self-talk are likely involved in mind wandering, mindfulness, self-concept clarity, and self-regulation in a variety of ways. Verbal self-directed thought, whether internal or external, may be beneficial or less helpful for self-regulation, depending on the context or valence. That is, self-talk to control emotions, thoughts, and behavior is likely helpful, but self-talk in a critical, negative fashion, as intuition might tell us, may not help the self to regulate. Mindful acceptance and self-concept clarity, which self-talk may be involved in, are also potentially helpful avenues to self-regulation, where mind wandering (also potentially involving self-talk) may be less so. 

One important distinction not discussed here is between self-reflection and self-rumination, where the former constitutes a healthy curiosity toward the self and the latter a repetitive and uncontrollable focus on negative aspects of the self [61]. Past work has addressed this distinction (e.g., [62,63]). Self-reflection has been shown to be associated with beneficial qualities (e.g., Theory-of-Mind), whereas self-rumination has been linked to anxiety and depression. Current efforts are aimed at carefully examining what the two opposite forms of self-focus entail (e.g., [64,65,66]).

There are limitations in this study beyond the sample characteristics (e.g., majority female, university students); for example, the questionnaires do not capture the actual frequencies of inner experiences (see [67]); rather, they capture the participants’ attitudes about or perceived frequencies of experiencing something related to the construct. To illustrate this, the GISQ [22] is not meant to measure the frequency of inner speech but rather the participants’ perceived frequencies of content, functions, and activities in general that may have involved inner speech. Moreover, the GISQ still requires refining of additional emergent factors and validity checks. Additionally, not only may some questionnaires be built on a priori notions about the nature of psychological constructs, but participants can have a priori or misinformed notions about constructs as well as the nature of their own minds. Moreover, those who report experiencing inner speech vary widely in their responses, and researchers do not yet know if everyone experiences inner speech [20]. There is an unfortunate stigma attached to “being caught” in the act of talking to oneself, which may affect people’s awareness of their own experience and their willingness to disclose. Even if self-talk is healthy, this does not mean that a lack of self-talk is unhealthy. People may leverage other forms of inner experience or states of mind in order to self-regulate, which may or may not be related to self-talk.

Our study was correlational in nature, and future research should aim to investigate the paths between these and other self-related constructs to see if variables such as participants’ pre-existing levels of self-concept clarity influence paths between inner speech and self-regulation. For example, one cannot infer that because mindful acceptance and self-concept clarity both relate to self-regulation, this means that practicing acceptance and gaining clarity causes increases in self-regulation. Factors such as cultural conceptions of the self (e.g., “I” versus “we” [5]), self-talk (e.g., [19]), or the way mindfulness is defined (e.g., with or without acceptance) may influence results. Future studies should also focus more on the temporal and developmental contexts of these self-processes. 

## Figures and Tables

**Table 1:** Present study hypotheses of mixed (M), positive (P), and negative (N) relationships.

H	Description	Literature Example(s)
H1	Self-talk and self-regulation—M	[19,44]
H2	Self-talk and self-concept clarity—P	[19,45]
H3	Self-talk and mindful awareness—P	[30]
H4	Self-talk and mindful acceptance—N	[30]
H5	Self-talk and mind wandering—P	[40,46]
H6	Self-concept clarity and self-regulation—P	[24,25]
H7	Mindfulness and self-regulation—P	[34,35]
H8	Mind wandering and self-regulation—N	[37]

Note: H = hypothesis number. Self-talk = out loud or internal mental speech about the self.

**Table 2:** Descriptive statistics and reliability of the study measures.

SCALE *Subscale* (α)	M	T	SD	Range	Min	Max	Skew	Kurtosis
GISQ (0.95)	209.6	285	35.77	180	105	285	−0.387	0.079
STS (0.88)	72.3	80	11.82	59	37	96	−0.203	0.018
*Social Assessment* (0.77)	18.3	20	3.92	18	6	24	−0.518	−0.238
*Self-Reinforcement* (0.88)	15.7	20	4.51	17	7	24	0.096	−0.739
*Self-Criticism* (0.84)	18.3	20	4.35	19	5	24	−0.535	−0.569
*Self-Management* (0.76)	19.9	20	3.22	15	9	24	−0.730	0.196
MWQ (0.84)	20.5	30	5.05	22	8	30	−0.156	−0.553
PHLMS *awareness* (0.79)	33.1	50	5.81	31	14	45	−0.366	−0.366
PHLMS *acceptance* (0.84)	23.2	50	7.46	32	10	42	0.303	−0.631
SCCS (0.87)	34.5	60	9.43	42	15	57	0.149	−0.614
SRQ (0.71)	214.6	325	20.87	128	147	275	−0.183	0.556

Note: M = mean; T = total possible; SD = standard deviation; Min = minimum observed score; Max = maximum observed score. Acronyms: GISQ = General Inner Speech Questionnaire; STS = Self-Talk Scale; MWQ = Mind Wandering Questionnaire; PHLMS = Philadelphia Mindfulness Scale; SCCS = Self-Concept Clarity Scale; SRQ = Self-Regulation Questionnaire.

**Table 3:** Correlation matrix representing basic relationships between the measures.

	1.	2.	3.	4.	5.	6.	7.	8.	9.	10.
1. STS Social Assess										
2. STS Regulation	0.21									
3. STS Criticism	0.56	0.21								
4. STS Management	0.68	0.30	0.49							
5. GISQ	0.55 *	0.44	0.41	0.52						
6. MWQ	0.26	0.02	0.22	0.18	0.23					
7. PHLMS	−0.09	0.10	−0.16 *	0.01	−0.09	−0.28				
8. PHLMS Aware	0.18	0.14 *	0.21	0.21	0.31	0.02	0.54			
9. PHLMS Accept	−0.29	0.03	−0.36	−0.18	−0.38	−0.39	0.71	−0.13		
10. SCCS	−0.28	0.07	−0.37	−0.17 *	−0.25	−0.37	0.35	−0.03	0.48	
11. SRQ	−0.16 *	0.21	−0.14 *	0.01	−0.05	−0.48	0.43	0.20	0.40	0.48

Note: all correlation coefficients are significant at the *p* < 0.01 level unless otherwise stated with an * (*p* < 0.05) or grey font (insignificant). GISQ = General Inner Speech Questionnaire; STS = Self-Talk Scale; MWQ = Mind Wandering Questionnaire; PHLMS = Philadelphia Mindfulness Scale; SCCS = Self-Concept Clarity Scale; SRQ = Self-Regulation Questionnaire.

**Table 4:** Observed relationships in context with main study hypotheses for discussion.

H	Hypothesized Relationships	Observed Relationships and H Support
H1	Self-talk and self-regulation—M	Mixed—supported
H2	Self-talk and self-concept clarity—N	Insignificant to N—mostly supported
H3	Self-talk and mindful awareness—P	P—supported
H4	Self-talk and mindful acceptance—N	Insignificant to N—mostly supported
H5	Self-talk and mind wandering—P	Insignificant to P—mostly supported
H6	Self-concept clarity and self-regulation—P	P—mostly supported
H7	Mindfulness and self-regulation—P	P—supported
H8	Mind wandering and self-regulation—N	N—supported

Note: the hypotheses are in the context of significance or non-significance. H = hypothesis number. Self-talk = out loud or internal mental speech about the self. M = mixed relationship; N = negative relationship; P = positive relationship (assuming significance).

## Data Availability

Data is available upon request.
